# Lifestyle Modification to Promote Weight Loss in the Absence of Energy Restriction

**DOI:** 10.1155/2011/527290

**Published:** 2011-10-02

**Authors:** Andrea C. Buchholz, Marta Van Loan, Leah Whigham, Henry Lukaski

**Affiliations:** ^1^Department of Family Relations and Applied Nutrition, University of Guelph, Guelph, ON, Canada N1G 2W1; ^2^Western Human Nutrition Research Center, ARS, USDA, University of California-Davis, Davis, CA 95616, USA; ^3^Grand Forks Human Nutrition Research Center, ARS, USDA, Grand Forks, ND 58202-9034, USA; ^4^Department of Physical Education, Exercise Science and Wellness, The University of North Dakota, Grand Forks, ND 58202-8235, USA

With the obesity epidemic showing no signs of abating, there is ongoing interest in altering energy balance (i.e., decreasing energy intake and/or increasing energy expenditure) to promote weight, specifically fat, loss. However, short- and long-term outcomes of, and adherence to, decreasing energy intake are variable, and reports of weight regain are common. There is thus increasing interest, both at the lay public and scientific levels, in lifestyle modifications to promote weight loss in the absence of energy restriction. In this special issue, we present two review papers and three randomized control trials that address such lifestyle modifications.

The first paper is a review of weight control strategies and the effects of these strategies on the hormonal responses associated with appetite control and regulation. A. Schwarz et al. conclude that consumption of smaller and more frequent meals comprised of lower fat and moderate protein, normal sleep of 8 hours/day, and control of stressors and psychological stress may help to attenuate the hormonal responses associated with appetite control. This is followed by the review of B. N. Wu and A. J. O'Sullivan, in which the authors stress the importance of considering gender differences in energy metabolism. This conclusion arises from the observation that compared to men, women consume fewer calories per kilogram lean mass and burn fat more preferentially during exercise, suggesting that the relationship between energy consumed and energy expended is different between the genders. The authors attribute the greater fat mass observed in women to ovarian hormones, particularly estrogen, which may promote postprandial conversion of dietary energy into body fat. From these two reviews, we appreciate the influence of lifestyle modification and gender on various aspects of energy balance. This understanding may in turn culminate in strategies to control or reverse fat gain beyond those that emphasize only energy restriction. 

In line with the dietary recommendations presented in the review of A. Schwarz et al., L. Ferguson-Stegall et al., in the first of three studies presented as part of this special issue, compared the effects of three experimental beverages: low-fat chocolate milk, an isocaloric carbohydrate beverage and a placebo beverage on training adaptation (cycling) of 32 untrained nonobese men and women. After each daily training session, the participants consumed their assigned experimental beverages immediately and again 1 hour after exercise. After 4.5 weeks, the differentials in fat mass loss and lean mass gain at the whole body and trunk levels were greater for the chocolate milk group versus carbohydrate group. The authors attribute this observation to the availability of amino acids in milk for anabolism and muscle mass gain, and the fat-loss-promoting effect of dairy calcium and protein, consistent with Schwarz and colleagues' recommendation for consumption of moderate protein and lower fat foods.

The next study, by S. L. Tey et al., examined the effects of daily isocaloric consumption of hazelnuts, chocolate, or potato chips for 12 weeks, compared to a control group receiving no snacks. Participants were 118 nonobese men and women; they were not provided any dietary counselling nor were they instructed to change their diets in any way. Despite the finding of no main effect of treatment, a subanalysis of adherent participants revealed that a higher baseline BMI was associated with a lower waist circumference at 12 weeks in the nut group versus control group. Also, dietary quality improved significantly in the nut group. The authors conclude that nuts can be incorporated into the diet without adversely affecting body weight, while improving overall diet quality. We extend this conclusion with the suggestion that if this observation were to hold over a longer study duration, then nuts might help to reverse the fat gain seen with aging. It is also tempting to suggest that regular consumption of nuts may reduce abdominal obesity in people with higher BMIs, although this requires confirmation.

In the final study, M. H. Pedersen et al. randomized 78 overweight adolescent boys to consume bread with fish oil or vegetable oil for 16 weeks. Unlike the previous study, in this study participants were counselled to improve diet and exercise habits. No changes were observed in resting metabolic rate, lipid oxidation, leptin levels, or body composition. However, this does not rule out metabolic benefits of long-chain polyunsaturated fatty acids in adults or adolescents not undergoing their pubertal growth spurt, nor does it rule out the possibility that obese boys with more pronounced metabolic complications may respond more favourably to a fish oil intervention. The lifestyle intervention was successful in decreasing the boys' sugar consumption, but had no impact on physical activity level, which the authors suggest indicates that changing an obviously poor dietary habit is easier than committing to increasing physical activity.

Given that weight loss success via energy restriction is difficult to achieve in the short term, with long-term success being even more elusive, novel approaches to control the obesity epidemic are warranted. Taken together, the five papers presented in this special issue support various lifestyle modifications to promote weight loss in the absence of energy restriction, although as with most diet-disease relationships, not consistently so. These modifications may include, but are not limited to, smaller and more frequent meals comprised of lower fat and moderate protein (e.g., dairy products and nuts, the latter of which are high in total fat that may not be highly bioavailable and are low in saturated fats), normal sleep of 8 hours/day, and control of stressors and levels of psychological stress. Further research is required to confirm these associations at various stages of the lifecycle, particularly in obese people.

**Andrea C. Buchholz** **Andrea C. Buchholz** **Marta Van Loan** **Marta Van Loan** **Leah Whigham** **Leah Whigham** **Henry Lukaski** **Henry Lukaski**

